# miR-31 and its host gene lncRNA LOC554202 are regulated by promoter hypermethylation in triple-negative breast cancer

**DOI:** 10.1186/1476-4598-11-5

**Published:** 2012-01-30

**Authors:** Katarzyna Augoff, Brian McCue, Edward F Plow, Khalid Sossey-Alaoui

**Affiliations:** 1 Department of Molecular Cardiology, Lerner Research Institute, Cleveland Clinic, Cleveland, OH, USA; 2 Department of Molecular Cardiology, Cleveland Clinic Lerner Research Institute, 9500 Euclid Ave., NB-50, 44195 Cleveland, OH, USA

**Keywords:** miR-31, LOC554202, Triple-negative breast cancer, lncRNA, CpG hypermethylation, Invasion-metastasis cascade

## Abstract

**Background:**

microRNAs have been established as powerful regulators of gene expression in normal physiological as well as in pathological conditions, including cancer progression and metastasis. Recent studies have demonstrated a key role of miR-31 in the progression and metastasis of breast cancer. Downregulation of miR-31 enhances several steps of the invasion-metastasis cascade in breast cancer, i.e., local invasion, extravasation and survival in the circulation system, and metastatic colonization of distant sites. miR-31 exerts its metastasis-suppressor activity by targeting a cohort of pro-metastatic genes, including RhoA and WAVE3. The molecular mechanisms that lead to the loss of miR-31 and the activation of its pro-metastatic target genes during these specific steps of the invasion-metastasis cascade are however unknown.

**Results:**

In the present report, we identify promoter hypermethylation as one of the major mechanisms for silencing miR-31 in breast cancer, and in the triple-negative breast cancer (TNBC) cell lines of basal subtype, in particular. miR-31 maps to the intronic sequence of a novel long non-coding (lnc)RNA, LOC554202 and the regulation of its transcriptional activity is under control of LOC554202. Both miR-31 and the host gene LOC554202 are down-regulated in the TNBC cell lines of basal subtype and over-expressed in the luminal counterparts. Treatment of the TNBC cell lines with either a de-methylating agent alone or in combination with a de-acetylating agent resulted in a significant increase of both miR-31 and its host gene, suggesting an epigenetic mechanism for the silencing of these two genes by promoter hypermethylation. Finally, both methylation-specific PCR and sequencing of bisulfite-converted DNA demonstrated that the LOC554202 promoter-associated CpG island is heavily methylated in the TNBC cell lines and hypomethylated in the luminal subtypes.

**Conclusion:**

Loss of miR-31 expression in TNBC cell lines is attributed to hypermethylation of its promoter-associated CpG island. Together, our results provide the initial evidence for a mechanism by which miR-31, an important determinant of the invasion metastasis cascade, is regulated in breast cancer.

## Background

Metastasis is responsible for ~90% of deaths in patients with solid tumors [1-4], including those originating in the breast [5-7]. Metastasis has always been portrayed as the ultimate step of the progressing breast cancers. Recent evidence, however, indicates that about a third of women diagnosed with small asymptomatic breast tumors (~4 mm) already harbor disseminated BC cells in their bone marrow [8]. Moreover, these micrometastases can remain dormant for years before reemerging as incurable secondary tumors and surprisingly insensitive to adjuvant chemotherapies that were originally effective against the primary tumor [9,10]. Adding to this problem is the fact that BC is a heterogeneous disease comprised of at least 5 genetically distinct subtypes, which together are the second leading cause of cancer deaths in women in the United States [11-13]. Within BC subtypes, those classified as Triple-Negative BCs (TNBCs) exhibit dismal survival rates due to their highly aggressive and metastatic behavior, and to their propensity to rapidly recur [14-20]. The TNBC subtype is characterized by lack of expression of hormone receptors (ER-α and PR) and HER2, harbor BRCA1-defects and/or deficiencies, and remain p53-positive [14,16-20]. Moreover, the absence of novel therapies capable of specifically targeting this very aggressive TNBC subtype reflects in part a lack of sufficient knowledge about TNBC development and progression [2,4,9,21].

microRNAs (miRs) are small noncoding RNAs, usually 20- to 22-nucleotides long, which regulate gene expression at the post-transcriptional level. To date, close to 1000 human miRs have been identified, which are thought to regulate more than 50% of human genes. miRs are now widely regarded as the most powerful regulators of gene expression in complex cellular processes including cancer cell invasion and metastasis [1,22-25]. In fact, several miRs, miR-15a, miR-16-1, and let-7 [23,26-30] function as tumor suppressors, and others, miR-155, miR-17-5p, and miR-21 [23,31,32], possess oncogenic properties

Several recent reports have identified a major role of miR31 in cancer metastasis [33-36] With regard to BC, we reported that miR-31 expression is lost in aggressive basal-type breast cancer cell lines compared to the non-invasive luminal counterparts. This observation was extended to human breast cancer tumors where we found an inverse correlation between miR-31 expression levels and advanced stages of BC [24]. Also, in our previously published work, we reported a highly significant correlation between the expression levels of WAVE3 and advanced stages of BC [37], supporting the function of WAVE3 as a metastasis promoter protein [38-40]. Linking these observations, we found that miR-31 regulated WAVE3 expression and activity during the invasion-metastasis cascade [24,25]. However, the upstream mechanisms of transcriptional regulation of miR-31 are not well understood and are the focus of the present study.

A recent study has predicted miR-31 to be transcribed from within the first intron of a host gene, LOC554202, on human chromosome 9 [41]. Our in silico analyses have confirmed these findings and suggest that LOC554202 is transcribed into a long non-coding RNA, (Lnc)RNA. We also identified a major CpG island upstream of the miR-31 locus, which also spans the first exon of LOC554202, suggesting an epigenetic regulation by methylation of both miR31 and its host gene. Here, we report that the expression pattern of miR-31 follows that of LOC554202 in the TNBC basal versus the luminal BC cell lines, supporting the hypothesis that miR-31 is under the transcriptional regulation as LOC554202. Next we show that loss of miR-31 expression in the aggressive TNBC cell lines is a direct consequence of hypermethylation of its associated promoter which also regulates LOC554202, the host gene for miR-31. Using both methylation-specific PCR (MSP) and bisulfite-modified DNA sequencing, we directly demonstrate that the miR-31 promoter is heavily methylated in basal TNBC compared to luminal BC cell lines. Our results not only identify a novel mechanism for miR-31 regulation but also clearly support the important role of promoter methylation in the suppression of miR-31 during tumor progression.

## Results

### miR-31 is transcribed from within the intronic sequence of a long non-coding (lnc)RNA, LOC554202

**Figure 1:**
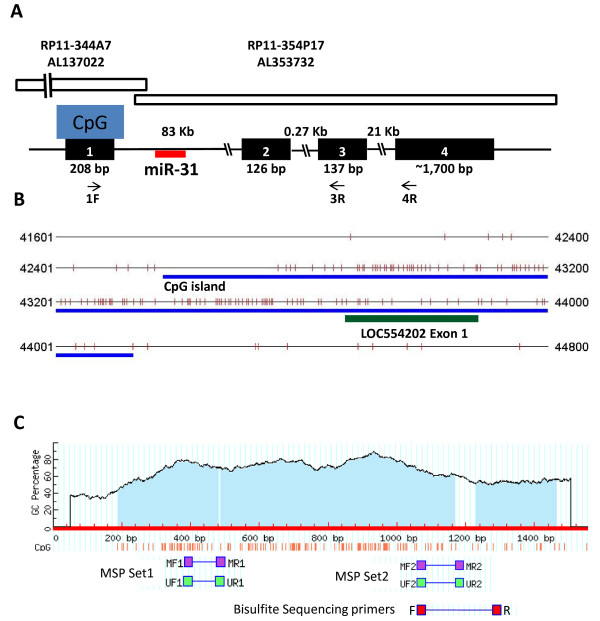
**Genomic organization of LCO554202/miR-31 locus and their associated CpG island**. **A**) Schematic representation of the genomic organization of the LOC554202 gene. BAC clones RP11-344A7 and RP11-354P17 are shown with horizontal open bars. A strong CpG island spans exon 1 of LOC554202. miR-31 which maps within intron 1 of LOC554202 is shown with a red horizontal bar. The size of each exon and intron is also shown. The arrows below Exon 1, Exon 3 and Exon 4 represent the location and orientation of the primers used for RT-PCR of LOC554202. **B**) The location of the predicted CpG island (blue line) and LOC554202 exon 1 (green line) are shown with respect to the sequence of Human BAC clone RP11-344A7 on chromosome 9p21.2-22.3, Accession number AL137022, which contains the 5' end of the LOC554202, the host Gene of microRNA miR-31. Each vertical bar represents one CpG dinucleotide. Exon 1 of LOC554201 is shown as a red line. **C**) Schematic representation of the predicted CpG island and the location of the MSP primers sets and the bisulfite sequencing primers.

miR-31 maps to BAC clone RP11-344A7 (Accession number AL137022) on human chromosome 9, which also contains 3' end of a newly identified LncRNA, LOC554202 (Figure 1A and Ref [41]). Our in silico analyses that determined the location of miR-31 within the first intron of LOC554202 also predicted a very strong CpG island in its associated promoter, upstream of miR-31 and spanning the first exon of LOC554202 (Figure 1B). Based on these observations, we hypothesized that (*i*) LOC554202 is the host gene for miR-31; (*ii*) as such, the transcriptional regulation of miR-31 follows that of its host gene LOC554202; and (*iii*) because of the presence of a strong CpG island associated with the promoter of LOC554202, both miR-31 and LOC554202 are epigenetically regulated by promoter methylation. Experiments were performed to test these predictions.

**Figure 2:**
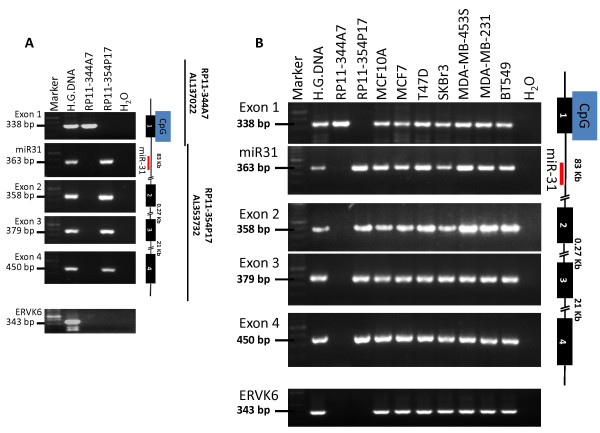
**The genomic structure of the LOC554202 miR-31 is preserved in the BC cell lines used in this study**. **(A) **Schematic representation of the mapping of the LOC554202 exons, the CpG island and miR-31 with respect to BAC clones RP11-344A7 and RP11-354P17. The mapping was confirmed by genomic PCR analysis. The size of the PCR product of each amplicon is shown on the left. ERVK6 was used as a positive control for the total genomic DNA. **(B) **Genomic PCR analysis of each exon of LOC554202 and miR-31 in the BC cell lines used in this study. The size of the PCR product of each amplicon is shown on the left. ERVK6 was used a positive control for the total genomic DNA.

The complete sequence of the processed LOC554202 transcript is 2246-bp long and is transcribed from 4 exons, based on two independently validated NCBI nucleotide sequences, accession number AK124393 and accession number NR_027054 [42,43](Ota et al., 2004 and Strausberg et al., 2002). Furthermore, according to Ensembl database (ensemble.org), the size of the LOC554202 gene is more than 106 kb (http://useast.ensembl.org/Homo_sapiens/Location/View?db=otherfeatures;g=554202;r=9:21454267-21559697;t=NR_027054.1), and spans two contiguous BAC clones on human chromosome 9: RP11-344A7 (Accession number AL137022) and RP11-354P17 (Accession number AL353732). Exon 1 and part of intron 1 map to BAC clone RP11-344A7, while the remaining sequence of intron 1, miR-31 and exon 2 to exon 4 map to BAC clone RP11-354P17. A schematic representation of the LOC554202 and miR-31 loci is depicted in Figure 2, panel A (Figure 2A). We used genomic DNA PCR to confirm the mapping of the 4 exons of LOC554202 as well as miR-31 to their respective BAC Clones (Figure 2A). As predicted, exon 1 was amplified only from BAC clone RP11-344A7 but not from BAC clone RP11-354P17, while miR-31 and exon 2, 3 and 4 were amplified from RP11-P17 but not from BAC clone RP11-344A7 (Figure 2A).

We also used genomic PCR analysis to show that all 4 exons of LOC554202 and miR-31 can be amplified from the genomic DNA of each of breast cancer cell lines we used in this study (Figure 2B). We, therefore, confirmed the integrity of the LOC554202 gene in these cell lines, and ruled out gross genomic alterations as a possible mechanism for the regulation of expression of both LOC554202 and miR-31.

### miR-31 and its host gene LOC554202 are down-regulated in TNBCs

**Figure 3:**
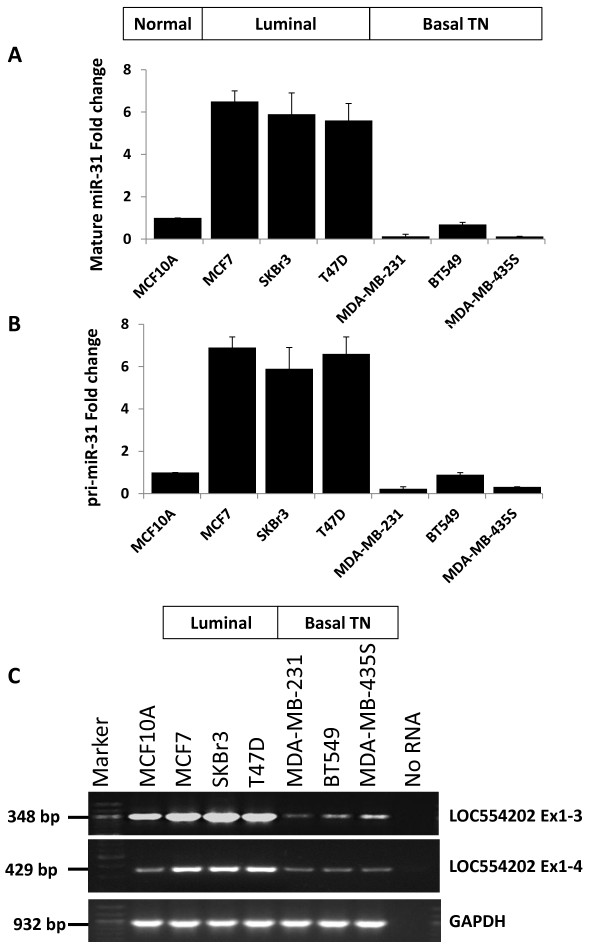
**miR-31 and its host gene LOC554202 are downregulated in basal TNBC cell lines and highly expressed in luminal non-invasive BC cell lines**. **(A and B) **Quantitative real-time RT-PCR of the mature miR-31 (A) and the pri-miR-31 (B) in non-malignant breast epithelial MCF10A, luminal MCF7, SKBr3 and T47D, and basal triple-negative MDA-MB-435S, MDA-MB-231 and BT549 BC cell lines. miR-16 and RNUB6 were used for normalization. **(C) **Semi-quantitative RT-PCR of the LOC554202 transcript in the same cell lines. GAPDH was used an internal control.

As a first step, we tested the relationship between expression levels of miR-31 and LOC554202 in a series of BC cell lines. We had previously shown that miR-31 is suppressed in the MDA-MB-231 cell line, an aggressive triple-negative BC of basal subtype, while it is expressed abundantly in the non-aggressive luminal subtype MCF7 cells [24]. We sought to determine whether this relationship extended to other BC cell lines of luminal versus basal subtypes. We found a very significant contrast in the expression profile of miR-31 between the luminal and basal BC cells. While the mature miR-31 is highly expressed in luminal BC subtypes, i.e., MCF7, SKBr3 and T47D cell lines, its expression is significantly reduced in the triple-negative basal subtypes such as MDA-MB-231, BT549 and MDA-MB-453S cell lines (Figure 3A). Similar trend was found for pri-miR-31, the precursor transcript for the mature miR-31 (Figure 3B). These data indicate that the loss of miR-31 associates with the aggressive TNBC cell lines. The expression profile of LOC554202 mirrors that of miR-31 in these same cell lines (Figure 3C); LOC554202 is expressed at significantly lower levels in the TNBC cell lines compared to the luminal counterparts.

### miR-31 and its host gene LOC554202 are epigenetically regulated in the TNBCs

**Figure 4:**
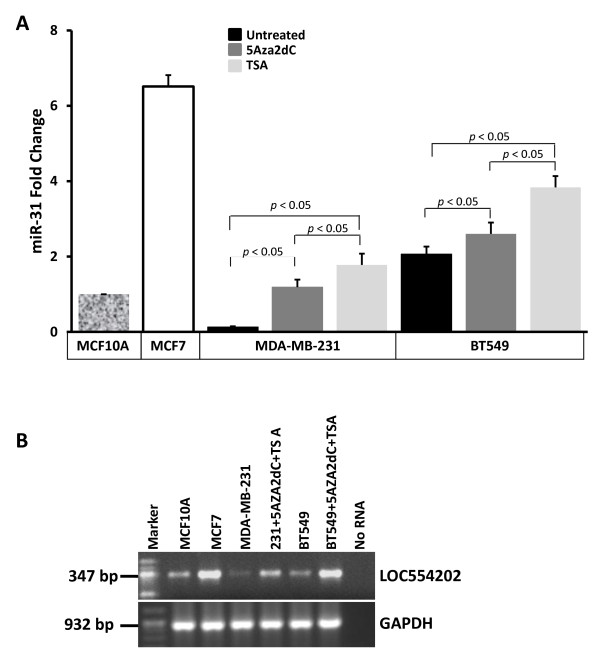
**miR-31 and LOC554202 are epigenetically regulated in breast cancer**. **(A) **Quantitative real-time RT-PCR of miR-31 in the indicated cell lines before and after treatment with 5 Aza-2dC and TSA. miR-16 was used for normalization. **(B) **Semi-quantitative RT-PCR of LOC554202 after treatment of MDA-MB-231 and BT549 with the de-methylating 5Aza2dC and the de-acetylating Trichostatin A (TSA) agents. Expression of LOC554202 in the untreated cell lines is also shown. GAPDH was used as an internal control.

The presence of a strong CpG island at the LOC554202-associated promoter suggests that transcription of both this gene and miR-31 may be regulated by methylation of the LOC554202-associated promoter. We therefore treated breast cancer cell lines, where expression of these two genes is down-regulated, with a de-methylating agent alone or in combination with a de-acetylating agent and assessed if expression of both LOC554202 and miR-31 was rescued. Treatment of both MDA-MB-231 and BT549 cells, which express low levels of either LOC554202 or miR-31, with the de-methylating agent 5Aza2dC resulted in a significant increase in the levels of both miR-31 (Figure 4A) and LOC554202 (Figure 4B). When these two cell lines were treated with a combination of both 5Aza2dC and the de-acetylating agent TSA, the expression levels of both genes increased to levels even higher than those observed with treatment with the de-methylating agent alone (Figure 4). These results clearly demonstrate an epigenetic regulation of both the LOC554202 and miR-31 by DNA methylation and probably by chromatin acetylation as well.

### CpG island methylation plays an important role in silencing of both the LOC554202 and miR-31 genes

**Figure 5:**
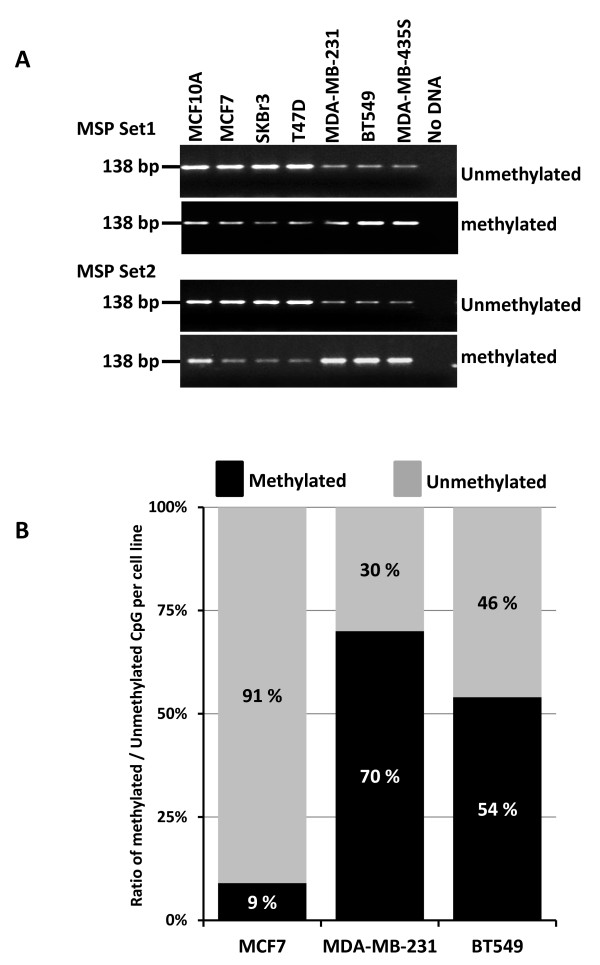
**miR-31 promoter is heavily methylated in the basal TN versus the luminal BC cell lines**. **(A) **Methylation specific PCR [MSP] of bisulfite-modified DNA of the indicated cell lines using 2 sets of primers. **(B) **Ratio of overall methylated over unmethylated CpGs by sequencing of bisulfite-modified DNA from the indicated cell lines. The location of the MSP and bisulfite sequencing primers is shown in Figure 1**C**.

To confirm the involvement of promoter methylation as a mechanism for gene silencing of both LOC554202 and miR-31, we used both methylation specific PCR (MSP) and sequencing of bisulfite-modified DNA to assess the methylation status of the CpG island in the LCO554202 promoter. MSP of two different regions of the CpG island (Figure 1C) showed that, while a strong PCR product was amplified from the non-malignant breast epithelial line MCF10A and the luminal BC subtypes MCF7, SKBr3 and T47D cell lines, using the unmethylation-specific primers, a PCR product of significantly lower intensity was detected in the TNBC MDA-MB-231, BT549 and MDA-MB453S cell lines (Figure 4A). The opposite results were obtained when we used methylation-specific primers, where a significantly strong PCR product was amplified from the cell lines of TNBC origin compared to the luminal BC subtypes (Figure 4A). Thus, the CpG island associated with the LOC554202 promoter is hypermethylated in the cell lines with low or no expression of either LOC554202 or miR-31 and is hypomethylated in the BC cell lines which express both genes at high levels. We further provided independent confirmation of the methylation status of the LOC554202-assocaited CpG island by sequencing of bisulfite-modified DNA from MCF7, MDA-MB-231 and BT549. In the MCF7 cell line where both the host gene LOC554202 and miR-31 are abundantly expressed, the ratio of methylated to unmethylated nucleotides for the total number of CpG dinucleotides surveyed was 9/91 (9% methylated and 91% unmethylated) (Figure 5B). On the other hand, in MDA-MB-231 and BT549 cell lines which express very low levels of either LOC554202 or miR-31, the methylated/unmethylated ratio was 70/30 and 54/46, respectively. Thus, the MSP data together with bisulfite sequencing demonstrate that loss of expression miR-31 and its host gene LOC554202 in the TNBC can be explained, at least in part, by hypermethylation of their promoter-associated CpG island, and thereby identifies a novel mechanism for the upstream regulation of miR-31 in TNBC.

## Discussion

Altered expression of microRNAs is frequently observed in human cancer, including ones originating in the breast [44-46], but the mechanisms underlying their regulation are poorly understood. We and others have previously shown that miR-31, a BC metastasis suppressor gene, is a major contributor to BC progression and metastasis by regulating a cohort of a pro-metastatic target genes, including WAVE3 [24], RhoA, Radexin [36] and several integrin subunits [47] that regulate key steps in the invasion-metastasis cascade. miR-31 expression levels are high in early stage BC tumors, allowing for its pro-invasive, pro-metastatic target genes to remain under tight control [24,36]. Expression levels of miR-31diminish as the tumors progress to more invasive stages [24] and become undetectable in metastatic BC tumors [36]. Loss of miR-31 expression is accompanied by concomitant increase in expression of its pro-invasive and pro-metastatic target genes, therefore, allowing for the tumor to become more invasive and ultimately metastasizes [24,36]. In BC mouse models, re-expression of miR-31 in the triple-negative MDA-MB-231 BC cells, which do not express endogenous miR-31, almost completely inhibits the metastatic potential of these cells without affecting the growth of the primary tumors while specifically inhibiting its pro-metastatic target genes ([36] and Sossey-Alaoui, unpublished data). On the other hand, knockdown of miR-31 in the non invasive luminal MCF7 BC cells results in lifting the inhibitory effect imposed by miR-31 on its target genes and imparts aggressive and metastatic phenotype to these cells comparable to observed in MDA-MB-231 cells [24,36]. Together, these published studies clearly demonstrate the important role that miR-31 plays during the invasion-metastasis cascade of BC tumors.

The mechanisms for upstream regulation of miR-31 leading to its loss during the invasion-metastasis cascade has been heretofore unknown. In this study, we report the contribution of epigenetic modifications as a novel mechanism by which miR-31 is regulated in breast cancer. First we showed that miR-31 is transcribed from the intronic sequence of LOC554202, a newly identified lncRNA. While both miR-31 and its host gene LOC554202 are expressed abundantly in the non-invasive BC cell lines of luminal subtype, their expression is lost in more aggressive TNBC cell lines of basal subtype, clearly suggesting that the transcription regulation of miR-31 might be under the control of its host gene LOC554202. Second, we identified a strong CpG island in the LOC554202-associated promoter, prompting us to hypothesize that both miR-31 in its host gene might be regulated by promoter methylation. Indeed, we were able to enhance expression of both miR-31 and LOC554202 in the TNBC cell lines after treatment with either the methylase inhibitor 5Aza2Cd alone or in combination with the acetylase inhibitor TSA. To further confirm the contribution of promoter hypermethylation to the loss of miR-31 in the TNBC cell lines we performed both methylation specific PCR and sequencing of bisulfite-modified DNA from both luminal (MCF7) and basal TNBC (MDA-MB-231 and BT549) cell lines. The combined number of CpG dinucleotides surveyed by these two assays allowed coverage of at least one third of total length of the CpG island. We found that while the LOC554201-associated promoter was significantly hypermethylated the in basal TNBCs, it was significantly hypomethylated in the luminal counterpart, further confirming that miR-31 expression is regulated in the TNBCs at least in part by promoter methylation. It is well established that hypermethylation of CpG islands associated with specific genes increases during the growth and progression of the primary tumor, providing a mechanism to inactivate tumor suppressor genes, DNA repair genes, cell cycle regulators and transcription factors. Based on our RT-PCR results, treatment of the TNBC cells with 5Aza2Cd or in combination with TSA enhanced expression of both miR-31 and its host gene LOC554202 to levels similar to those found in luminal BC subtypes. The restoration of miR-31 expression by these maneuvers was also very significant but did not reach the levels observed in luminal BC (Figure 3). One possible explanation for this difference is that, in addition to promoter methylation that regulates both miR-31 and LOC554202, other mechanisms may selectively regulate miR-31.

It is worth noting that our study appears to be the first to report that LOC554202 might belong to the lncRNA family. Our preliminary in silico analyses show that the LOC554202 locus spans more that 100 kilobases (kb) of genomic sequence and that its RNA is transcribed from 4 exons resulting in a spliced transcript of ~2.2 kb (Figure 1), which does not contain an open-reading frame that could be translated into a functional protein, and therefore can be classified a lncRNA. It is possible that this new lncRNA may have a function in chromatin remodeling and epigenetic regulation of gene expression similar to that of the well known XIST and HOTAIR lncRNAs [48,49]. More experimental analyses are however required to investigate the exact function of LOC554202 in breast cancer invasion and metastasis.

## Conclusion

The present study, although conducted in established cell lines, clearly identifies promoter hypermethylation as a novel mechanism by which miR-31 is silenced during the invasion-metastasis cascade of BC. Future studies using biological specimens with associated clinico-pathological parameters and disease outcome, are required to further confirm these findings and to assess whether miR-31 promoter methylation can be used a prognostic marker for BC progression and survival outcome.

## Methods

### Cell lines and their treatment

Human non-malignant breast epithelial cell line MCF10A and the human breast cancer cell lines were obtained from American Type Culture Collection (Rockville, MD). Cells were cultured at 37°C with 5% CO_2 _in their specified basic culture medium supplemented with 4.5 g/L glucose, 10% fetal bovine serum (Invitrogen), 2 mmol/L glutamine and antibiotics. The demethylating agent 5Aza2dC (Sigma, MO, USA) was freshly prepared in double-distilled H_2_O and filter sterilized. Cells (5-10 × 10^5^) were seeded in a 100 mm tissue culture dish in culture medium at 37°C, 10% CO_2_. The next day, cells were treated with 0.5 μM of 5aza2dC. The culture medium containing the demethylating agent was replaced every day for 7 days. For the 5Aza2dC-Trichostatin A (TSA, Sigma, MO, USA) dual treatment, TSA (0.3 μM final concentration) was added to the culture at day 5 for a 48-h treatment period. At the end of the treatment period, total RNA was prepared using TRIzol (Invitrogen, CA, USA), according to the manufacturer's instructions. The BAC clones were obtained from the Rowell Park Cancer Institute BAC Library and BAC DNA was isolated using the the QIAprep Spin Miniprep kit (Qiagen Sciences, MD, USA). The total genomic DNA was prepared using proteinase K digestion as previously described [50].

### Semi-quantitative and Real-time quantitative-PCR

Total RNA was extracted from cancer cell lines using TRIzol reagent (Invitrogen, Carlsbad, CA), following to the manufacturer's instructions. cDNA was generated and used as a template for semi-quantitative RT-PCR performed as previously described [25,37,51,52]. Expression levels of the precursor (pri-miR) and the mature forms of microRNA miR-31 were quantified by real-time quantitative RT-PCR using human TaqMan MicroRNA Assays Kits (Applied Biosystems, Carlsbad, CA). We used GAPDH to normalize the expression levels of LOC554202 transcripts. In addition, we found that both miR-16 and RNU6B were expressed at similar levels in all cell lines analyzed, when normalized to GAPDH (Additional File 1). Furthermore, treatment of BC cells with either 5Aza2dC, Trichostatin A or both, did not affect their expression levels when compared to the untreated cells (Additional File 1), and therefore, were used for normalization of miR-31 expression levels across the breast cancer cell lines and between treatments. The reverse transcription reaction was carried out with TaqMan MicroRNA Reverse Transcription Kit (Applied Biosystems, Carlsbad, CA) following the manufacturer's instructions. Quantitative PCR was performed on the BioRad (Hercules, CA) MyiQ2 iCycler PCR system where the reaction mixtures were incubated at 95°C for 10 min, followed by 40 cycles of 95°C for 15 s and 60°C for 1 min. The cycle threshold (Ct) values were calculated with SDS 1.4 software (Bio-Rad). The expression levels of miR-31 were normalized using the 2^-ΔΔCt ^method [53] relative to miR-16 or RNU6B. The ΔCt was calculated by subtracting the Ct values of miR-16 from the Ct values of miR-31. The ΔΔCt was then calculated by subtracting ΔCt of each breast cancer cell line from MCF10 cells. Fold change in the gene expression was calculated according to the equation 2^-ΔΔCt^.

### Preparation of bisulfite-modified DNA for methylation analysis

Genomic DNA (1-2 μg) was denatured in 0.3 M NaOH for 30 min at 42°C, and then the unmethylated cytosine residues were sulphonated by incubation in 3.12 M sodium bisulfite (pH 5.0; Sigma)/5 mM hydroquinone (Sigma, MO, USA) at 55°C for 16 h. The sulphonated DNA was recovered using the QIAquick Gel Extraction system (Qiagen, MA, USA), according to the manufacturer's recommendations. The conversion reaction was completed by desulphonating in 0.3 M NaOH for 5 min at room temperature. The DNA was ethanol precipitated and resuspended in double-distilled water.

### CpG island prediction and primer design for methylation analysis

The LOC554202 putative promoter region was predicted from the genomic sequence of BAC clone RP11-344A7, accession number AL137022, for the sequence around its first exon using the PromoterInspector prediction software (http://www.genomatix.ed). The promoter-associated CpG island was predicted using the CpG prediction algorithm (http://www.urogene.org/methprimer/help.html#cpg_prediction), and primers for sequencing of bisulfite-modified DNA and for methylation specific PCR (Additional File 2) were designed using the Methprimer algorithm (http://www.urogene.org/methprimer/index.html).

### Sequencing of bisulfite-modified DNA

In total, 20-50 ng of bisulfite-treated DNA was used as template in each PCR reaction under the following conditions: 95°C for 5 min, followed by 40 cycles of 15 s of denaturation at 95°C, 20 sec at 55°C and 25 sec of extension at 72°C. The PCR reaction was terminated with an additional 7 min of extension and cooled to 4°C. The PCR products were resolved on a 2% agarose gel, stained with ethidium bromide, and the 250-bp bands were excised and gel-purified using the QIAquick Gel Extraction system (Qiagen, MA, USA). The purified PCR products were cloned into the pCR2·1-TOPO vector (Invitrogen, CA, USA), and at least 15 clones were sequenced from each cell line. The methylation status at each CpG site was analyzed using the MethTools software (http://genome.imb-jena.de/methtools/). The overall methylation status in each cell line was calculated as a ratio of the number of unmethylated to methylated CpGs and plotted as a percentage of total number of CpGs analyzed.

### Methylation specific PCR

Methylation specific PCR (MSP) was performed on bisufite-converted DNA using the MSP primer pairs described in Additional file 2. Each DNA sample was PCR-amplified using either the methylated or the unmethylated primer pairs. The PCR products were next resolved by agarose electrophoresis, stained with ethidium bromide and a picture recorded. The intensities of the PCR products between the methylated and unmethylated primer-pairs were compared by densitometry.

### Oligonucleotide primer sequences

Sequences of the oligonucleotide primers used for genomic PCR, RT-PCR from IDT (San Diego, CA) and are listed Additional file 2.

### Statistical analyses

The data are presented as the means ± standard errors of at least three independent experiments. The results were tested for significance using an unpaired Student's *t *test and *p *values of < 0.05 were considered statistically significant.

## Abbreviations

WAVE3: Wisckott aldrich verprolin family member 3; BC: Breast cancer; TNBC: Triple-negative breast cancer; ER: Estrogen receptor; PR: Progesterone receptor; EMT: Epithelial to mesenchymal transition; miR: microRNA; cDNA: Complementary-DNA; BAC: Bacterial artificial chromosome; PCR: Polymerase chain reaction; RT-PCR: Reverse transcriptase PCR; qt-rt-RT-PCR: Quantitative real-time RT-PCR; MSP: Methylation specific PCR; TSA: Trichostatin A.

## Competing interests

The authors declare that they have no competing interests.

## Authors' contributions

KA carried out most of the experiments including the quantitative real-time RT-PCR for miR-31 and LOC445202 as well as the methylation specific PCR and assisted in drafting the manuscript. BM performed the bisufite sequencing experiments and was responsible for the tissue culture of cell lines and their treatments as well as DNA and RNA isolation. EFP discussed the design of the experiments, the results and assisted in writing. KSA supervised the overall design of the experiments and wrote the manuscript.

### Grant support

DOD Breast Cancer Idea Award grant W81XWH0810236 (K. Sossey-Alaoui), NIH grants P01HL073311 and P50HL077107 (E.F. Plow). K. Augoff is funded in part by a research fellowship "Development program of Wroclaw Medical University" from the European Social Fund, Human Capital, national Cohesion Strategy" (contract # UDA-POKL.04.01.01-00-010/08-00)".

## Supplementary Material

Additional file 1 **Quantitative real-time RT-PCR of miR-16 (A and B) and RNU6B (C and D) in the indicated cell lines as well as before and after treatment with 5Aza-2dC and TSA**. GAPDH was used as an internal control for normalization. Click here for file

Additional file 2 **List of oligonucleotide primers and microRNA assays used in this study**. Click here for file
