# Supplementary figures and images for: miR-31 and its host gene lncRNA LOC554202 are regulated by promoter hypermethylation in triple-negative breast cancer
Source: Mol Cancer. 2012 Jan 30;11:5. doi: 10.1186/1476-4598-11-5 (PMC3298503; doi:10.1186/1476-4598-11-5)

**A**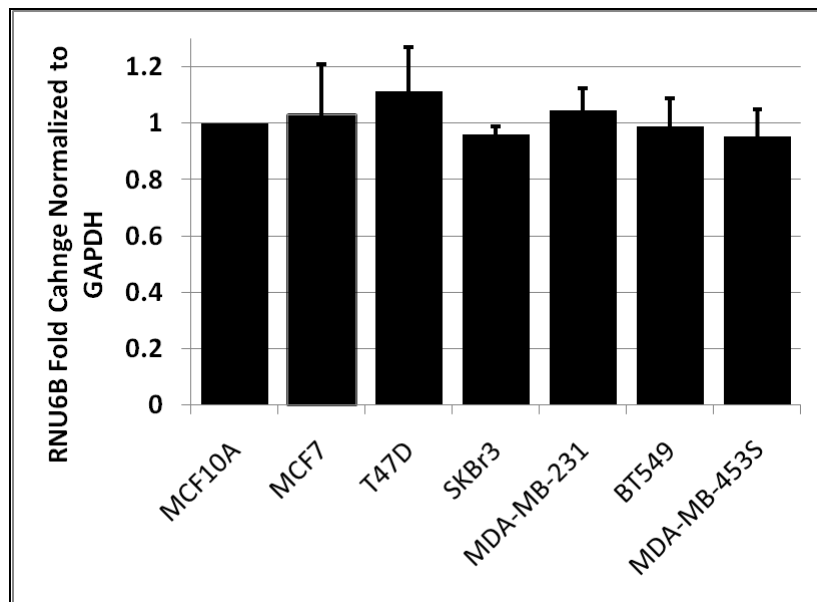**B**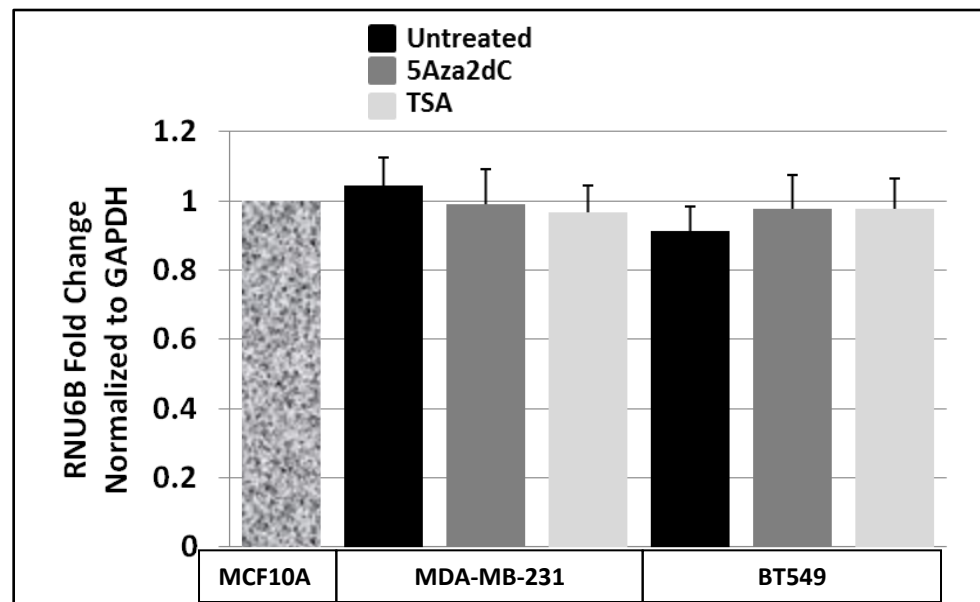**C**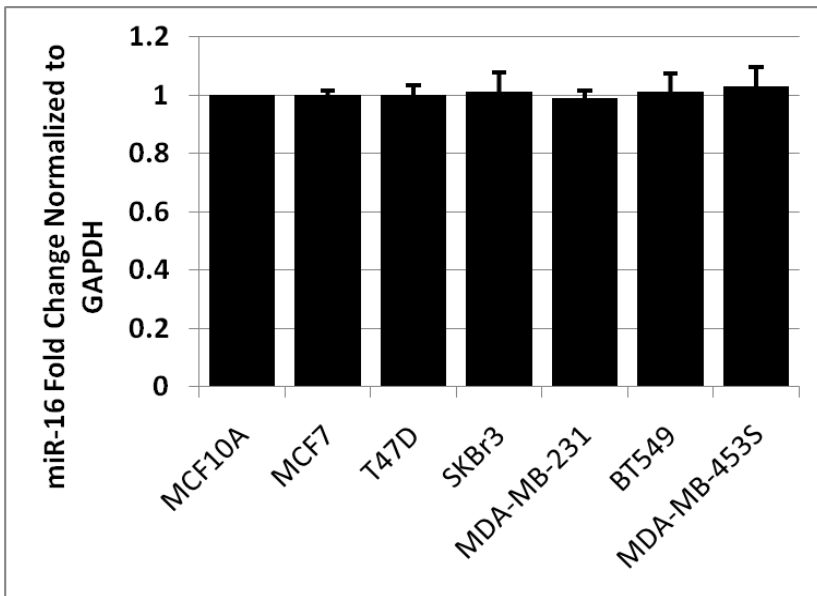**D**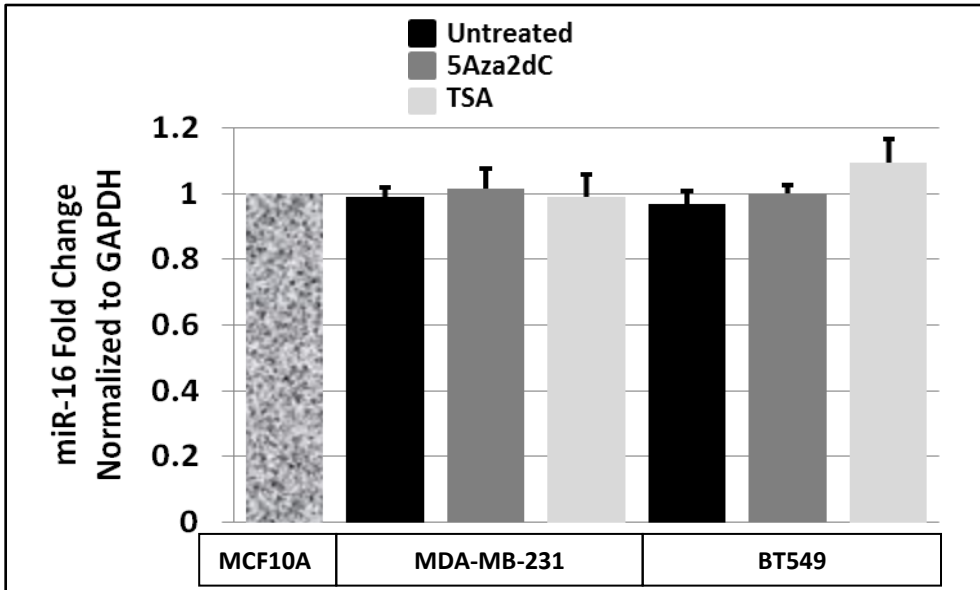

Supplement: Additional file 1 — Quantitative real-time RT-PCR of miR-16 (A and B) and RNU6B (C and D) in the indicated cell lines as well as before and after treatment with 5Aza-2dC and TSA. GAPDH was used as an internal control for normalization. [file 1476-4598-11-5-S1.PDF]
