# Supplementary material for: miR-31 and its host gene lncRNA LOC554202 are regulated by promoter hypermethylation in triple-negative breast cancer
Source: Mol Cancer. 2012 Jan 30;11:5. doi: 10.1186/1476-4598-11-5 (PMC3298503; doi:10.1186/1476-4598-11-5)
Supplement: Additional file 2 — List of oligonucleotide primers and microRNA assays used in this study. [file 1476-4598-11-5-S2.PDF]

Additional file 1: List of oligonucleotide primers and microRNA assays used in this study.

Oligonucleotide primers used for Genomic PCR of the LOC554202 locus

LOC554202Ex1GF: 5’-GCAGCTGCGACCTGTGCATAACTT-3’
LOC554202Ex1GR: 5’-GGCCCCGAAGCCTCCTCAACTC-3’
PCR product size: 338 bp

LOC554202Ex2GF: 5'-TTTTTCTATCACTGCCTTTTCACA-3’
LOC554202Ex2GR: 5’-GCCCCCAACTCTATTTACCAA-3’
PCR product size: 358 bp

LOC554202Ex3GF: 5’-GTGAAAAATGGCAAACTTAT-3’
LOC554202Ex3GR: 5’-GACAACAAATTCTGAGATGAGGAT-3’
PCR product size: 379 bp

LOC554202Ex4GF: 5’-TAGGGCTGCCAGTAGAGGGAAGAG-3’
LOC554202Ex4GR: 5’-GCAAGCAGGCCAACCAACAAG-3’
PCR product size: 450 bp

LOC554202Ex4GF: 5’-TAGGGCTGCCAGTAGAGGGAAGAG-3’
miR-31-Genomic-R: 5’-CATCTTCAAAAGCGGACACTCTAAGGAAGACTATGTTG-3’
PCR product size: 363 bp

ERVK6-F: 5’-AGGGACTAGGGAAAAATGAAGATG-3’
ERVK6-R: 5’-GGGTTGAATTACGGCGTTTACAGC-3’
PCR product size: 343 bp
Maps to BAC clone RP11-33P21 from human chromosome 7

Oligonucleotide primers used for Bisulfite sequencing

Forward primer: 5’-GGGATTTAGGTTTTTTTATTGTAA-3’
Reverse Primer: 5’-AAAAAACCCCTAAAAAAACAAAAAT-3’
Number of CpG dinucleotides covered: 14
Product size: 250 bp

Oligonucleotide primers used for Methylation–Specific PCR

MSP set 1
Left M primer 1: 5’-CGGGATTTAGGTTTTTTTATTGTAAC-3’
Right M primer 1: 5’-CCTCTCCCTTAACTCTAACTACGAA-3’
Left U primer 1: 5’-TGGGATTTAGGTTTTTTTATTGTAATG-3’
Right U primer 1: 5’-CCTCTCCCTTAACTCTAACTACAAA-3’
Product size: 138 bp

Additional File 1 Continued

MSP set 2
Left M primer 2: 5’-CGGGATTTAGGTTTTTTTATTGTAAC-3’
Right M primer 2: 5’-CCTCTCCCTTAACTCTAACTACGAA-3’
Left U primer 2: 5’-TGGGATTTAGGTTTTTTTATTGTAATG-3’
Right U primer 2: 5’-CCCTCTCCCTTAACTCTAACTACAAA-3’
Product size: 138 bp

Oligonucleotide primers used for RT-PCR

LOC554202 Ex1 F: 5’-CAGAGCTGGGAGGCGGTGTTC-3’
LOC554202 Ex3R: 5’-AGGAGGCTGGGAGGGTGGTCT-3’
LOC554202 Ex4R: 5’- CTCTACACTGGCCTTGAGGAGGTA-3’

GAPDH-F: 5'-TGAAGGTCGGAGTCAACGATTTGGT-3'
GAPDH-R: 5'-CATGTGGGCCATGAGGTCCACCAC-3'
PCR product size: 932 bp

miR-31 transfection reagents

miR-31 mature sequence: AGGCAAGAUGCUGGCAUAGCU
pre-miR-31 precursor: Ambion Product Number: AM17100; Product ID: PM11465
Negative Control miR: Ambion Product Number: AM17110

TaqMan microRNA assays for qt-RT-PCR

Mature miR-31 TaqMan microRNA Assay ID 002279
Pri-miR31 microRNA TaqMan assay ID Hs03302684_pri
miR-16 TaqMan microRNA Assay ID 000391
RNU6B TaqMan microRNA Assay ID 001093
